# Personalizing Maintenance Rituximab in Follicular Lymphoma: A Machine Learning Framework for Risk–Benefit Optimization

**DOI:** 10.34133/hds.0467

**Published:** 2026-08-12

**Authors:** Junyi Gao, Jiaxin Liu, Xinze Li, Meng Wu, Rongyi Cui, Yannan Huang, Zhixin Zhang, Yinghao Zhu, Yasha Wang, Yuqin Song, Ewen M. Harrison, Lan Mi, Liantao Ma, Yan Xie

**Affiliations:** ^1^ Key Laboratory of Carcinogenesis and Translational Research (Ministry of Education/Beijing), Department of Lymphoma, Peking University Cancer Hospital & Institute, Beijing, China.; ^2^ Centre for Medical Informatics, University of Edinburgh, Edinburgh, UK.; ^3^ Health Data Research UK, London, UK.; ^4^ National Engineering Research Center for Software Engineering, Peking University, Beijing, China.; ^5^ Department of Automation, Tsinghua University, Beijing, China.

## Abstract

**Background:** While maintenance rituximab therapy (MT) for follicular lymphoma improves progression-free survival, individual benefit varies, creating a critical need to avoid overtreatment and its associated burdens. **Methods:** We developed a double machine learning framework using a retrospective 404-patient cohort to estimate the individualized treatment effect of MT on the risk of disease progression within 24 months. **Results:** Our model revealed heterogeneity in treatment benefit, successfully distinguishing patients most likely to benefit from those who are not. A retrospective simulated application identified a “low-risk, low-benefit” subgroup that might potentially forgo MT. Furthermore, aligning historical clinical decisions with our model’s recommendations was associated with a lower progression rate compared to nonalignment (14.8% vs. 38.0%). Extensive sensitivity analyses confirmed the robustness of this treatment heterogeneity against potential unmeasured confounding and temporal practice shifts. **Conclusions:** Supported by an updated interactive clinical decision tool, this data-driven framework provides a robust strategy to personalize MT by separating prognostic risk from predictive benefit. It serves as a hypothesis-generating template to guide future prospective risk-adapted trials and reduce unnecessary treatment in oncology.

## Introduction

Follicular lymphoma (FL) outcomes have improved with immunochemotherapy, yet management remains contentious, particularly regarding maintenance rituximab therapy (MT). While MT following induction therapy prolongs progression-free survival, it exposes patients to excessive treatment, financial burden, and a higher risk of infection [1,2]. This paradox has created a critical clinical dilemma: a “one-size-fits-all” recommendation for 2 years of MT is standard for responders [3], yet there is widespread uncertainty about which individuals truly benefit. Some patients may be overtreated, while others might require more intensive strategies. This uncertainty is reflected in practice variation and trials like the Rituximab Extended Schedule Or Retreatment Trial (RESORT) [4], which showed that rituximab re-treatment at relapse could achieve similar disease control with less drug exposure, underscoring the central question: can we move beyond population-average benefits to precisely identify which patients need maintenance therapy [4]?

Traditional prognostic scoring systems, such as the Follicular Lymphoma International Prognostic Index (FLIPI), stratify patients based on parameters such as age, disease stage, and blood counts to help predict survival rates [5,6]. The most widely used such marker is progression of disease within 24 months (POD24), which identifies a high-risk cohort with dismal survival [7]. However, a fundamental limitation persists: these tools are prognostic, not predictive. They can estimate a patient’s likely outcome but cannot predict the magnitude of benefit that the patient will derive from a specific treatment, such as MT. A patient’s baseline risk may not correlate with their responsiveness to therapy [8]. Consequently, we lack validated tools to prospectively guide the MT decision for an individual, leaving a major gap in clinical management that risk-adapted trials are now seeking to address [9,10].

To bridge this gap from prognosis to prediction, we require methods that can estimate the individualized treatment effect (ITE)—the benefit that a specific patient is predicted to receive from a therapy compared to an alternative. Recent advances in machine learning, particularly methods adapted from causal inference, provide a framework to estimate such effects from observational data [11,12]. By systematically accounting for confounding factors, these models aim to disentangle the impact of the treatment itself from the patient’s underlying prognosis. This approach moves beyond identifying who is at high risk to pinpointing who is most likely to respond to an intervention, forming a more robust evidence base for personalized risk–benefit decisions.

**Fig. 1.**
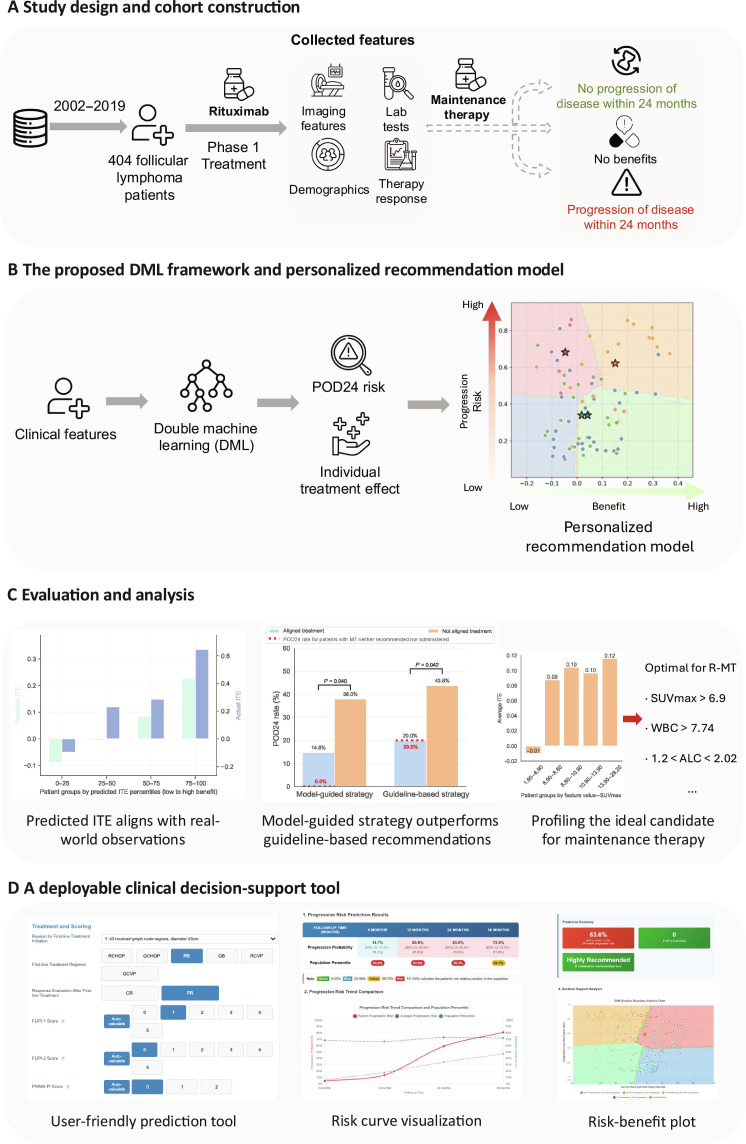
The proposed machine learning framework to personalize maintenance rituximab therapy in follicular lymphoma. (A) Study design and cohort construction: A retrospective cohort of 404 patients with follicular lymphoma who received first-line immunotherapy was established. Comprehensive clinical data, including demographics, imaging features, lab tests, and therapy response, were collected. The primary outcome was progression of disease within 24 months (POD24) following the decision to administer or withhold maintenance rituximab therapy (MT). (B) The proposed double machine learning (DML) framework and personalized recommendation model: Patient clinical features were input into a DML model to disentangle prognostic risk from predictive benefit. The model generates 2 personalized outputs for each patient: the baseline risk of POD24 and the individualized treatment effect (ITE) of MT. These 2 outputs are then integrated into a personalized recommendation model, visualized as a risk–benefit matrix that stratifies patients into distinct subgroups. (C) Framework evaluation and analysis: The model was validated and analyzed on the test set. (Left) The model’s predicted ITE showed strong alignment with observed real-world treatment effects. (Center) The model-guided recommendation strategy was associated with better clinical outcomes than a standard guideline-based approach, notably by identifying a patient group that may potentially forgo MT with 0.0% progression. (Right) The model enables personalized profiling for the ideal therapy candidate. For example, patients with a higher SUVmax at diagnosis derive the greatest benefit from MT. (D) A deployable prediction and result visualization tool: To facilitate clinical implementation, we developed an interactive decision-support tool to allow clinicians to make predictions and visualize results efficiently. The application will also collect clinicians’ predictions and evaluations for future human–artificial intelligence (AI) alignment research and prospective studies.

Here, we develop and validate a machine learning framework to personalize MT decisions for patients with FL (Fig. 1). Using a large, single-center retrospective cohort of 404 patients, we estimate the ITE of MT on preventing POD24 for each individual. We then integrate these ITE estimates with baseline risk predictions to construct a personalized decision-support framework. Critically, we validate this framework through a simulated clinical application, demonstrating that aligning treatment with our model’s recommendations is associated with a lower rate of disease progression compared to those of both nonalignment and standard guideline-based approaches. This work provides a data-driven strategy to optimize the risk–benefit trade-off of MT, offering a path to minimize overtreatment and advance the paradigm of personalized therapy in lymphoma. Finally, to translate this framework into clinical practice, we developed and present an interactive web-based decision-support tool designed to bring these personalized predictions to the point of care (available at https://fl-ite.medx-pku.com).

## Methods

### Study design and cohort construction

**Fig. 2.**
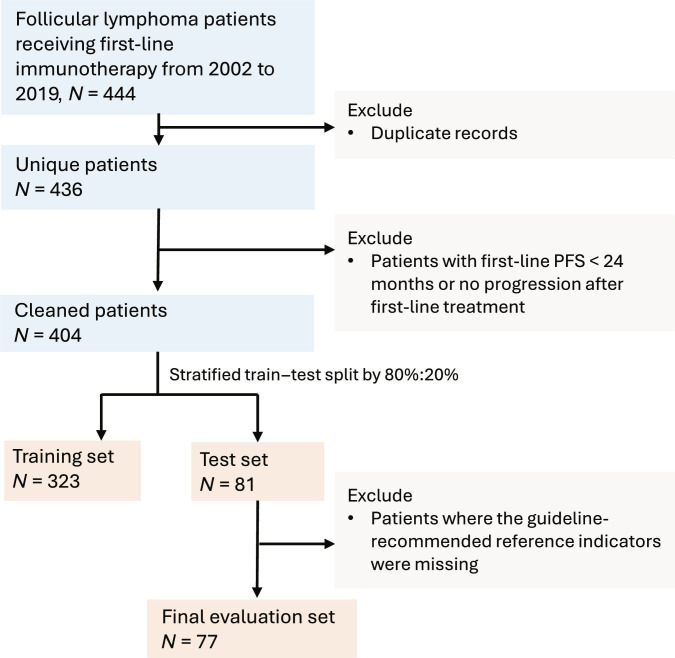
Data cleaning and split strategy.

The study cohort was retrospectively assembled from patients diagnosed with FL who received first-line immunochemotherapy between 2002 and 2019. The initial dataset comprised 444 records. After removing duplicate entries and data cleaning, a cohort of 404 patients was established, following established practices for leveraging fine-grained electronic medical records in longitudinal clinical studies [13]. This cleaned dataset was then partitioned using a stratified 80%:20% split to create a training set (*N* = 323) and a test set (*N* = 81). A final filtering step was applied to the test set to exclude patients for whom guideline-recommended reference indicators were not available, yielding a final evaluation set of 77 patients (Fig. 2).

The selection of clinical predictors for the machine learning models was determined by 3 primary criteria: (a) established prognostic significance in FL, specifically variables comprising the FLIPI [14] and FLIPI-2 [5] scoring systems (e.g., age, clinical stage, hemoglobin, β2-microglobulin, longest diameter of the largest node, and bone marrow involvement); (b) routine availability in standard clinical practice, including baseline laboratory tests and imaging parameters such as SUVmax; and (c) variables demonstrating potential prognostic value or imbalances in univariate analysis (Table 1).

**Table 1.** Comparison of demographic and clinical variables between all patients, the POD24 = 1 cohort, and the POD24 = 0 cohort, showing means, standard deviations, and *P* values.

Name	Type ^a^	All		POD24 = 1		POD24 = 0		*P* value ^b^
		Mean	SD	Mean	SD	Mean	SD	
Reason for initiating first-line treatment	Categorical	ND		ND		ND		ND
Treatment classification	Categorical	ND		ND		ND		ND
Maintenance therapy (rituximab based)	Categorical-d	Positive 158 (39.1%)		Positive 34 (25.4%)		Positive 124 (45.9%)		0.083
Sex	Categorical-d	Female 215 (53.2%)		Female 61 (45.5%)		Female 154 (57.0%)		0.903
Response evaluation group	Ordinal	0.85	1.07	1.43	1.09	0.56	0.94	0.037*
Lymph node size >6 cm at treatment initiation	Ordinal-d	0.41	0.50	0.53	0.52	0.36	0.49	0.436
Bone marrow involvement at treatment initiation	Ordinal-d	0.47	0.51	0.54	0.51	0.43	0.50	0.754
Clinical stage at initial treatment	Ordinal	3.40	0.87	3.61	0.68	3.30	0.93	0.000*
B symptoms	Ordinal-d	0.27	0.50	0.40	0.60	0.20	0.43	0.000*
ECOG performance status	Ordinal	0.52	1.09	0.93	1.41	0.31	0.82	0.000*
Histopathological grade at first-line treatment	Ordinal	0.64	1.66	0.64	1.82	0.64	1.58	0.057
Lymphocyte-to-monocyte ratio (LMR)	Continuous	5.73	10.47	7.11	12.31	5.28	9.77	0.008*
Absolute lymphocyte count	Continuous	2.70	12.95	5.10	25.27	1.91	3.40	0.000*
Absolute monocyte count	Continuous	0.43	0.32	0.52	0.56	0.41	0.17	0.000*
Platelet count	Continuous	199.89	67.89	205.76	87.62	197.98	60.23	0.000*
Hemoglobin	Continuous	129.19	17.61	118.47	16.98	133.02	16.22	0.593
White blood cell count	Continuous	7.25	13.85	10.26	26.99	6.28	4.01	0.000*
β2-microglobulin	Continuous	3.23	6.92	3.69	1.50	3.02	8.28	0.000*
Lactate dehydrogenase	Continuous	231.00	132.66	266.43	155.67	214.14	116.79	0.000*
Number of involved nodal areas	Continuous	7.62	4.28	8.49	3.80	7.19	4.44	0.045*
Age	Continuous	49.07	12.44	49.02	12.94	49.10	12.21	0.429
SUVmax	Continuous	10.57	4.85	11.21	4.67	10.33	4.91	0.684
Maximum lesion diameter	Continuous	5.86	3.43	6.78	3.65	5.50	3.28	0.187

POD24, progression of disease within 24 months; ECOG, Eastern Cooperative Oncology Group

^a^ “-d” denotes dummy variable, which takes values of 0 or 1.

^b^ *P* values less than 0.05 are indicated by an asterisk (*), denoting statistical significance.

### Modeling framework for ITE estimation

To estimate the ITE of MT on POD24 from observational data while controlling for confounding, we employed a double machine learning (DML) framework [11]. The DML approach provides robust, debiased estimates of causal effects by using machine learning models to partial out the effects of covariates. The procedure was implemented in 2 main stages.

#### Stage 1: Nuisance parameter estimation

In the first stage, 2 auxiliary models (nuisance functions) were trained on the patient covariates X.

• Outcome model: A model was trained to predict the conditional expectation of the outcome given the covariates, $E\left[Y|X\right]$. This model estimates the baseline risk of POD24 for each patient, irrespective of the treatment received.
• Treatment model (propensity score): A second model was trained to predict the conditional probability of receiving MT given the covariates, $E\left[T|X\right]$. This model estimates the propensity score for each patient.

Both the outcome and treatment models were implemented using the CatBoost classifier algorithm [15], which is well suited for handling a mix of continuous and categorical features.

#### Stage 2: Treatment effect estimation

In the second stage, the ITE was estimated using the residuals from stage 1. First, the outcome residual ($Y_{\mathrm{res}}$) and treatment residual ($T_{\mathrm{res}}$) were calculated for each patient:

$$Y_{\mathrm{res}}=Y-\hat{E}\left[Y|X\right]$$ (1)

$$T_{\mathrm{res}}=T-\hat{E}\left[T|X\right]$$ (2)

where $\hat{E}\left[\cdot |X\right]$ represents the predictions from the stage 1 models. The ITE for each patient, denoted as $\theta \left(X\right)$, was then estimated by fitting a final-stage model to solve the following residual-on-residual regression:

$$Y_{\mathrm{res}}=\theta \left(X\right)\cdot T_{\mathrm{res}}+\epsilon$$ (3)

This final stage was implemented using a CatBoost regressor, which learned the function $\theta \left(X\right)$ by predicting the ratio $Y_{\mathrm{res}}/T_{\mathrm{res}}$ with a sample weight of $T_{\mathrm{res}}^{2}$. The resulting $\theta \left(X\right)$ represents the estimated treatment effect of MT on the probability of POD24 for a patient with covariates X. A positive value indicates a reduction in POD24 risk (benefit), while a negative value suggests no benefit or potential harm.

#### Cross-fitting

To mitigate overfitting and prevent biases that can arise from using the same data to estimate nuisance functions and the final treatment effect, we implemented a k-fold cross-fitting procedure. The training dataset (*N* = 323) was split into k=3 folds. For each fold, the stage 1 models were trained on the other k−1 folds and then used to generate predictions (and, thus, residuals) for the held-out fold. This process was repeated for all folds, ensuring that the residuals for every patient in the training set were generated from models that they were not trained on. The final-stage ITE model was then trained on the complete set of out-of-sample residuals from the entire training set.

### Development of the personalized clinical decision model

To translate the model outputs into an actionable clinical recommendation, we developed a decision-support tool. We used the 2 key personalized outputs from our DML framework—the predicted baseline risk of POD24 (from the outcome model) and the estimated ITE (from the final-stage model)—as features for a second-level classifier. A support vector machine (SVM) with a radial basis function kernel was trained to classify patients into 1 of 4 strata based on these 2 inputs: (a) low-risk/low-benefit, (b) low-risk/high-benefit, (c) high-risk/low-benefit, and (d) high-risk/high-benefit.

All analyses were conducted using Python (version 3.8), with the “scikit-learn”, “pandas”, and “CatBoost” libraries.

### Simulated clinical application and comparison with guidelines

To assess the potential clinical utility of our framework, we designed and conducted a retrospective analysis simulating its application in our cohort.

#### Recommendation strategy

Based on the SVM classification, we formulated a recommendation strategy aimed at maximizing benefit while minimizing overtreatment. The strategy advises maintenance rituximab for all patient subgroups except those classified as having both low progression risk and low predicted ITE benefit.

#### Evaluation framework

We evaluated this strategy by comparing disease progression rates based on whether the treatment that each patient actually received was aligned or not aligned with our model’s recommendations. As a benchmark, we performed the same alignment analysis using standard clinical guideline recommendations [16], with detailed criteria provided in Supplementary Materials A.1.

#### Interpretation of outcomes

Evaluating treatment recommendations is limited by the counterfactual problem—we observe only the outcome for the treatment received. Therefore, we assessed the plausibility of the recommendations by examining subgroups where the observed clinical outcome, given the actual treatment decision, appeared consistent or inconsistent with the recommendation’s underlying logic. These observable scenarios were categorized as follows:

• Scenarios suggesting that the recommendation was appropriate:
■ (Aligned: recommended MT, received MT, progressed): Progression occurred despite recommended MT, consistent with a high-risk patient for whom MT was warranted.
■ (Aligned: recommended against MT, no MT, no progression): No progression occurred when MT was withheld as recommended, supporting the recommendation’s validity.
■ (Not aligned: recommended MT, no MT, progressed): Progression occurred when the recommended MT was withheld, suggesting that following the recommendation might have prevented progression.
• Scenarios suggesting that the recommendation was potentially inappropriate:
■ (Aligned: recommended against MT, no MT, progressed): Progression occurred when MT was withheld as recommended, indicating a potential missed benefit from MT.

Groups with unobservable counterfactuals (e.g., patients who received MT and did not progress) were not used to directly evaluate the recommendation’s correctness. Differences in progression rates between groups were assessed using Fisher’s exact test.

### Target trial emulation framework

To strengthen the causal credibility of our observational study, we explicitly framed our dataset within a target trial emulation framework. “Time zero” was strictly defined as the clinical evaluation point immediately following the completion of first-line induction immunochemotherapy, at which the decision to initiate or withhold MT was made. This design eliminates immortal time bias by ensuring that patient characteristics were assessed prior to the MT decision, and the primary endpoint (POD24) was measured strictly within 24 months from this baseline.

### Statistical and sensitivity analyses

To address the inherent limitations of observational causal inference and small subgroup variances, we conducted several rigorous sensitivity analyses.

#### Propensity score overlap

We extracted propensity scores from the treatment model to visualize the distribution density between MT and non-MT groups. We restricted specific causal interpretations to the “common support” region to avoid unreliable model extrapolation.

#### Unmeasured confounding (E value)

We utilized the E-value methodology to quantify the minimum strength of an unmeasured confounder required to completely explain away the observed MT protective effect.

#### Nonparametric bootstrapping

To stabilize variance in small subgroups, we employed nonparametric bootstrapping with *B* = 2,000 resamples. This allowed us to compute robust 95% confidence intervals (CIs) for the area under the Qini curve (AUQC) and to statistically test the ITE differences between the highest- and lowest-benefit subgroups (stratified by both quartiles and tertiles).

#### Temporal stratification

To assess whether the learned heterogeneity merely reflected era-specific practice patterns over the nearly 2-decade study period, we stratified the cohort into 3 eras, early (2002 to 2010), mid (2011 to 2015), and late (2016 to 2019), evaluating the consistency of the treatment effect across time.

## Results

Our study sought to develop a personalized treatment strategy for maintenance rituximab (MT) in FL. We addressed 3 key research questions:

• Can we accurately predict a patient’s baseline risk of early progression (POD24)?
• Can we reliably estimate the ITE of MT?
• Can a decision model based on these estimates improve upon current guideline-based care?

### Patient cohort and baseline characteristics

Our study cohort comprised 404 patients with FL who received first-line immunochemotherapy at one institution 3. Baseline demographics and clinical characteristics are summarized in Table 1. Patients who experienced POD24 (*n* = 81, 20.0%) presented with higher-risk features at diagnosis compared to those who did not, including a more advanced clinical stage, presence of B symptoms, a higher Eastern Cooperative Oncology Group performance status, and a higher tumor burden as indicated by laboratory values (*P* < 0.05 for all). These differences underscore the established prognostic factors associated with early progression and represent the confounders that our model must account for.

### A robust prognostic model for predicting POD24

Accurately predicting patients at risk for early progression (POD24) represents a critical prerequisite step in our treatment effect estimation framework. The development of this prognostic model serves 2 essential purposes: first, to identify patient-specific risks associated with disease progression and, second, to establish a robust foundation for our subsequent causal inference analysis. The DML model allowed us to disentangle the true effect of maintenance therapy from confounding factors in our observational dataset. Using confounders to directly predict POD24 allows us to separate the treatment effect from the treatment strategy later, as detailed in the methods section.

On an independent test set, the resulting model demonstrated strong discriminative performance with an area under the receiver operating characteristic curve (AUROC) of 0.92 and an area under the precision–recall curve of 0.87, indicating excellent ability to distinguish between patients who would and would not experience POD24.

It is worth emphasizing that while this predictive model achieved substantial discriminative performance, absolute predictive accuracy is not a strict requirement for our subsequent causal inference methodology. The DML framework is designed to accommodate imperfect outcome prediction while still yielding unbiased treatment effect estimates. Nevertheless, the strong performance of our POD24 prediction model enhances the precision of our ITE estimates.

### Model reveals substantial heterogeneity in treatment benefit

Next, we implemented a DML model to estimate the ITE of MT for each patient. The ITE quantifies the predicted reduction in POD24 risk attributable to MT, with positive values indicating benefit. The challenge in estimating treatment effects from observational data lies in the counterfactual nature of individual outcomes—each patient can receive only one treatment strategy, making direct measurement of individual benefit impossible. Therefore, robust evaluation methods must rely on comparing outcomes across different patient subgroups. To validate these estimates, we stratified test set patients into quartiles based on their predicted ITE, creating 4 distinct subgroups from the lowest predicted benefit (0 to 25th percentile) to the highest predicted benefit (75th to 100th percentile). Each subgroup has 20 patients.

**Fig. 3.**
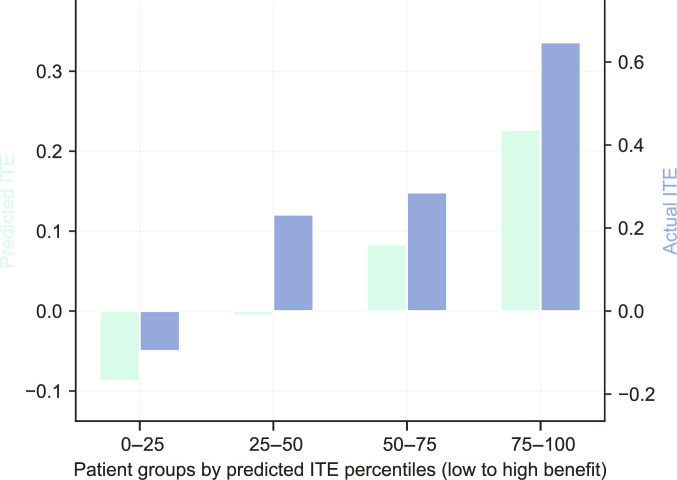
Comparison of predicted and observed individualized treatment effects (ITEs) across patient subgroups stratified by predicted ITE. Each group has 20 patients. Green bars represent the model-predicted ITE, while blue bars indicate the actual observed treatment effects within each subgroup. A monotonic trend between predicted and observed effects supports the discriminative power of the model.

A clear monotonic relationship emerged: patients predicted to benefit most also exhibited the largest observed treatment effect, while those predicted to benefit least showed no observed benefit (Fig. 3). Notably, the model identified a substantial subgroup of patients for whom MT was predicted to be beneficial (ITE > 0). However, for patients in the lowest-benefit quartile, the model predicted a neutral or even negative ITE, suggesting that MT would be ineffective in this population. This finding aligns with clinical observations that certain patients may experience adverse effects or paradoxical responses to prolonged immunotherapy [17].

**Fig. 4.**
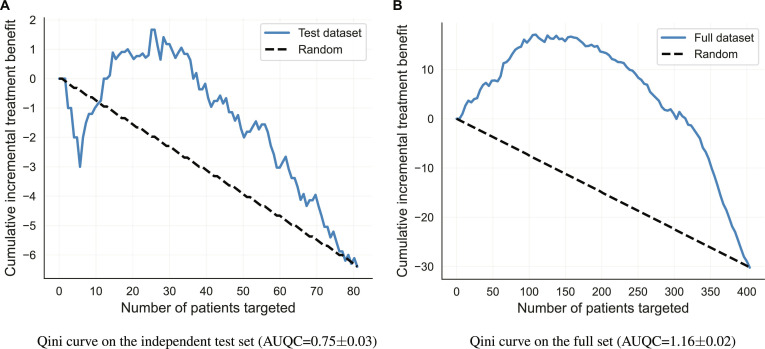
Qini curve of the model prediction on the test set (A) and training set (B). Standard deviations are calculated using nonparametric bootstrap with 1,000 resamples.

The model’s ability to separate responders from nonresponders was further confirmed by an AUQC of 0.75 on the test set (Fig. 4A), indicating strong performance in identifying patients who benefit most from treatment. The AUQC metric quantifies the model’s ability to identify patients most likely to benefit from the intervention, with values substantially above zero (random assignment) indicating effective treatment effect estimation. The initial dip in the curve on the test set is due to the high variance from the small sample size (fewer than 10); in this range, a single patient’s outcome can cause large fluctuations. However, the curve quickly recovers and shows a sustained positive lift, confirming the model’s robust performance in identifying patients who will benefit the most. These results demonstrate that the benefit of MT is highly heterogeneous and that our model can successfully identify this variation at an individual level.

### Simulated application of a personalized decision model suggests improved risk stratification compared to standard guidelines

**Fig. 5.**
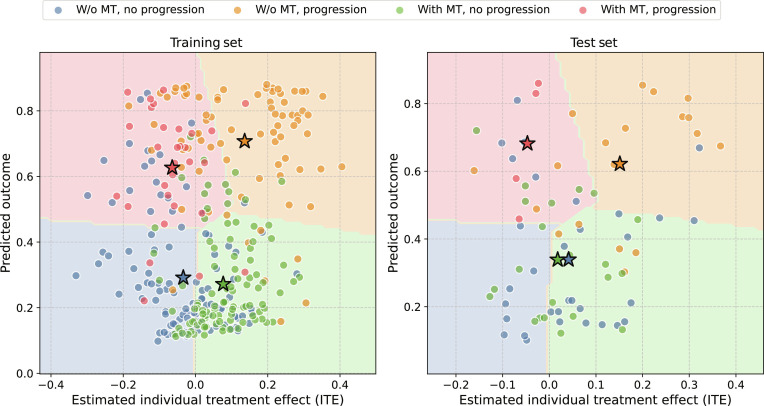
Visualization of estimated individual treatment effect (ITE) vs. predicted progression risk. Patients are plotted based on their estimated ITE (*x*-axis) and predicted progression risk (*y*-axis) for both the training set (left) and test set (right). Points are colored according to actual treatment received (MT = maintenance therapy) and observed progression status. Background shading represents the 4 patient subgroups derived from the support vector machine (SVM) classification based on risk and ITE. Stars indicate the centroids for each treatment/outcome group.

To translate our ITE and risk estimates into clinical action, we used an SVM model to stratify patients into 4 distinct risk/benefit groups (Fig. 5). We then formulated a recommendation strategy and retrospectively simulated its application, assessing outcomes based on alignment between the model’s recommendation and the actual treatment received (see the “Simulated clinical application and comparison with guidelines” section for details).

**Fig. 6.**
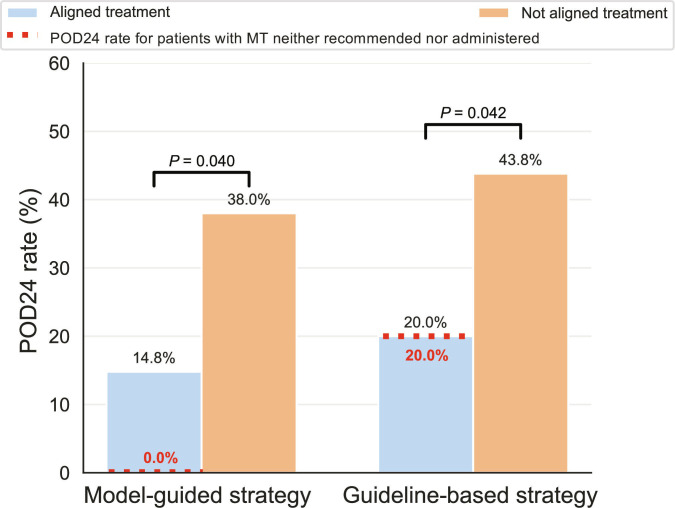
Model-guided recommendations are associated with a lower rate of disease progression. The chart shows progression of disease within 24 months (POD24) rates based on whether patient treatment was aligned with our model’s recommendations or standard guidelines. The model-aligned group had a lower progression rate than the guideline-aligned group (14.8% vs. 20.0%). Critically, among patients recommended to forgo maintenance therapy, the model-guided strategy was associated with a 0.0% progression rate, compared to 20.0% for the guideline-based strategy (red dashed line).

Our analysis revealed a marked difference in outcomes based on alignment with the model’s recommendations (Fig. 6 and Table 2). Patients whose actual treatment aligned with the model’s suggestions experienced a substantially lower overall progression rate (14.8%) compared to those whose treatment diverged (38.0%). This difference is statistically significant (Fisher’s exact test, *P* = 0.040), indicating that adherence to the model’s recommendations is associated with a significant reduction in disease progression in this cohort.

**Table 2.** Comparison of patient outcomes based on alignment with treatment recommendations. This table compares disease progression outcomes for patients whose actual treatment decisions were either aligned or not aligned with recommendations from our SVM classification model versus current clinical guidelines. The model categorizes patients by POD24 risk (high/low) and predicted benefit from maintenance rituximab (high/low) to generate personalized treatment recommendations. Bold groups are scenarios where we considered the recommendation as reasonable. Underlined groups are scenarios where we considered the recommendation as unreasonable or incorrect. Other groups are counterfactual outcomes.

Treatment decision alignment	Treatment received	Disease progression	No disease progression
Model-guided treatment recommendations			
Aligned with model	Maintenance rituximab	**4 (21.1%)**	15 (78.9%)
	Without maintenance rituximab	0 (0%)	**8 (100%)**
	Subtotal	4 (14.8%)	23 (85.2%)
Not aligned with model	Maintenance rituximab	0 (0%)	7 (100%)
	Without maintenance rituximab	**19 (44.2%)**	24 (55.8%)
	Subtotal	19 (38.0%)	31 (62.0%)
Current clinical guideline recommendations			
Aligned with guidelines	Maintenance rituximab	**4 (20.0%)**	16 (80.0%)
	Without maintenance rituximab	5 (20.0%)	**20 (80.0%)**
	Subtotal	9 (20.0%)	36 (80.0%)
Not aligned with guidelines	Maintenance rituximab	0 (0%)	6 (100%)
	Without maintenance rituximab	**14 (53.8%)**	12 (46.2%)
	Subtotal	14 (43.8%)	18 (56.2%)

SVM, support vector machine; POD24, progression of disease within 24 months

When compared to a benchmark of standard clinical guidelines, alignment with our model was associated with a lower progression rate than alignment with guidelines (14.8% vs. 20.0%), although this difference was not statistically significant (*P* = 0.755).

A crucial finding emerged when evaluating patients recommended to forgo maintenance therapy. The model’s strategy resulted in zero instances (0% of 8 patients) of progression among this group, whereas the guideline-based approach was associated with a 20.0% progression rate (5 of 25 patients) in the equivalent subgroup. While this stark contrast did not reach statistical significance (*P* = 0.302), likely due to small subgroup sizes, it represents a clinically vital observation, suggesting that the model may be more effective at identifying patients who may potentially forgo MT without increasing their risk of progression. It is important to emphasize that this alignment analysis is a retrospective construct driven by historical clinician behavior rather than a randomized exposure. Unmeasured clinical factors or quality-of-care differences may confound these results. Therefore, the observed association between model alignment and lower POD24 rates should be interpreted cautiously as descriptive and hypothesis generating, serving as a foundation for future randomized trials rather than definitive evidence of policy superiority.

### A clinical decision-support tool for personalized maintenance therapy

To translate our findings into a practical asset, we developed an interactive, web-based decision-support tool (Fig. 1D). This application makes our complex model accessible at the point of care, providing clinicians with individualized predictions to guide MT decisions. The online prediction tool is publicly available at https://fl-ite.medx-pku.com.

Upon entering a patient’s data, the tool delivers a clear, multilayered summary. It begins with an immediate assessment: the patient’s 24-month progression risk and treatment recommendation. For deeper insight, the tool provides the following:

• Personalized risk trajectory: This visualizes the patient’s progression risk over time and compares it to the population average, offering crucial context for patient conversations.
• Transparent benefit analysis: The core of the tool; this feature plots the patient on the risk–benefit map from our analysis (Fig. 5), making it transparent why a recommendation is being made. The ITE is also compared to the population average.
• Actionable feature insights: This shows which specific clinical factors are driving the prediction of benefit for that individual patient.
• FLIPI-2 benchmarking: The traditional FLIPI-2 score is automatically calculated and displayed prominently, allowing clinicians to instantly benchmark the DML model’s personalized prediction against a familiar population-level prognostic index.

This tool is designed not to replace clinical judgment but to augment it with data-driven, personalized evidence. To enable continuous validation, the application captures the clinician’s own assessment of risk and their treatment plan before revealing the model’s output and then records their level of agreement with the model’s recommendations. This framework will allow for rigorous human–model alignment evaluation and is a critical step toward our ultimate goal: initiating prospective trials to evaluate how this system can improve treatment quality and efficiency. By making the model’s reasoning transparent, the tool facilitates more evidence-based discussions and represents a tangible step toward implementing personalized medicine in the clinic.

### Disentangling prognostic risk from predictive benefit

**Fig. 7.**
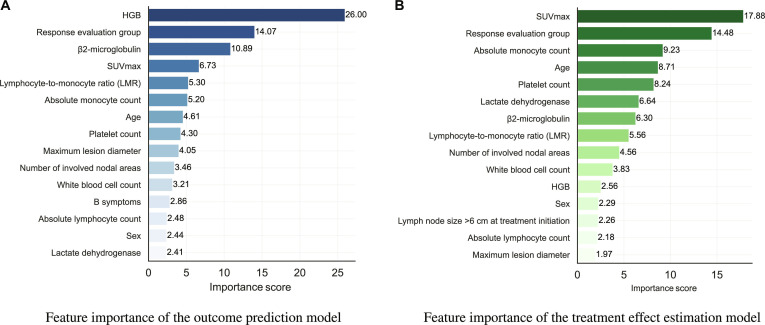
Feature importance of the outcome prediction model (A) and the treatment effect estimation model (B).

A central advance of our framework is its ability to separate the clinical features that drive a patient’s baseline prognosis from those that predict their response to MT. Our prognostic model confirmed the importance of established risk factors like hemoglobin and β2-microglobulin (Fig. 7A), which are primarily markers of disease burden and aggressiveness [5]. In stark contrast, our model, which predicted the benefit of MT, prioritized different factors (Fig. 7B). The 2 most influential predictors of treatment effect were SUVmax and the quality of response to induction therapy.

This distinction is clinically profound. It suggests that the greatest benefit from MT is derived by patients with biologically active disease (high SUVmax) who may have residual, metabolically avid tumor cells even after achieving a good response to induction. This aligns with the biological rationale for maintenance therapy: to eradicate minimal residual disease (MRD) that is still sensitive to anti-CD20 therapy [9]. The dual importance of features like SUVmax in both models highlights a complex interplay: high metabolic activity marks a patient as high-risk, but it also appears to mark them as a high responder to continued therapy. This nuanced insight is inaccessible to traditional prognostic scores and underscores the need for predictive modeling [18].

### Profiling the ideal candidate for maintenance therapy

**Fig. 8.**
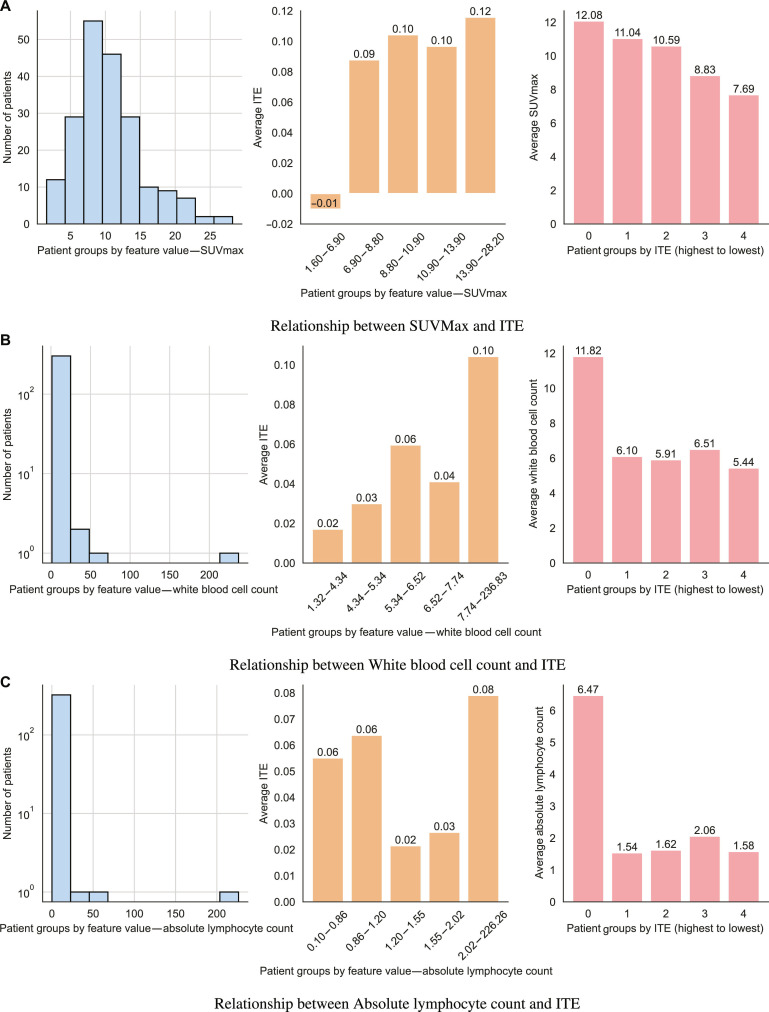
Relationship between patient feature values and individual treatment effect (ITE). This figure presents 3 visualizations exploring the association between a patient-specific feature and their ITE. (A) Number of patients across feature value buckets: Patients are grouped into 5 buckets based on their feature values (from lowest to highest). Each feature value bucket has the same number of patients. (B) Average ITE across feature buckets: The average ITE is calculated and displayed for each bucket, illustrating how ITE varies with different levels of the feature. (C) Average feature value across ITE quantiles: Patients are grouped into 5 quantiles based on their ITE (from highest to lowest). The average feature value is then calculated and displayed for each ITE quantile.

The ITE model allows us to create data-driven profiles of patients most and least likely to benefit from MT. Our analysis revealed a clear dose–response relationship between SUVmax and treatment benefit (Fig. 8A); patients with the highest metabolic tumor activity derived the largest benefit, while those with the lowest activity derived none. This provides quantitative evidence to support the clinical intuition that MT is most critical for patients with aggressive disease biology at diagnosis.

Furthermore, we identified intriguing associations with baseline immune parameters. Patients with higher white blood cell (>7.74, Fig. 8B) and absolute lymphocyte counts (>2.02, Fig. 8C) at diagnosis were predicted to gain more from MT. While correlational, this generates a testable hypothesis: a more robust baseline immune system and hematopoietic reserve may be necessary to mount an effective, sustained response to immunotherapy with rituximab. These feature-specific analyses transform the abstract ITE score into a set of clinically recognizable patient profiles, offering a tangible tool to guide shared decision-making at the bedside.

Collectively, these examples illustrate how the ITE model can be used as a tool to dissect the heterogeneity of treatment benefit. Rather than relying on a single risk score, clinicians could potentially use a combination of such individual patient characteristics to build a more nuanced picture of who is most likely to benefit from MT. These analyses transform prognostic markers into predictive tools, directly linking a patient’s baseline clinical features to their potential for therapeutic gain and helping to identify cohorts where MT is highly beneficial versus those where it may be potentially omitted.

### Robustness and sensitivity analyses

#### Validation of the target trial emulation (propensity score overlap)

To validate our target trial emulation framework and ensure that causal estimates were derived from a population with clinical equipoise, we evaluated the propensity score distribution between the MT and non-MT groups. The analysis revealed a well-defined “common support” region ranging from 0.40 to 0.65, capturing approximately 69.8% of the patient cohort. Sensitivity analysis demonstrated high stability: the average treatment effect (ATE) and discriminative performance in the full cohort (*n* = 424; ATE = −0.09, AUROC = 0.93) remained highly consistent when strictly limited to the common support region (*n* = 294; ATE = −0.07, AUROC = 0.91). This confirms that our ITE inferences are grounded in empirical overlap rather than algorithmic extrapolation (Fig. S2).

#### Bootstrap verification of treatment heterogeneity

To address variance in small subgroups, we performed nonparametric bootstrapping (*B* = 2,000). The global AUQC was remarkably robust at −24.33 (95% CI [−28.39, −19.71], cleanly excluding 0), confirming overall treatment effect heterogeneity (Fig. S3). The empirical benefit was profound in the highest-benefit quartile (Q1 benefit = −0.93, 95% CI [−0.99, −0.85]) and contrastingly absent or harmful in the lowest-benefit quartile (Q4 benefit = 0.58, 95% CI [0.39, 0.75]). The benefit difference between these extremes was highly significant across both quartile splits (difference: −1.50, 95% CI [−1.69, −1.31], *P* < 0.001) and tertile splits (difference: −1.19, 95% CI [−1.37, −1.00], *P* < 0.001), underscoring the statistical reliability of the identified subgroups (Fig. S4).

#### E-value sensitivity analysis

The overall median E value was exceptionally strong at 2.69 (interquartile range: 1.60 to 2.93), and the top 20% highest-benefit cohort achieved an E value of 3.86. For the Q1 benefit quartile, the conservative lower bound of the E value was 2.23. This indicates that an unmeasured confounder would need to be associated with both MT assignment and POD24 by a risk ratio of at least 2.23 (beyond measured covariates) to shift the CI to include the null, confirming high causal credibility. Conversely, for the lowest-benefit (Q4) subgroup, the group-level relative risk was 1.28 (95% CI [1.08, 1.37]); because no protective effect was observed (relative risk > 1), the E value is inherently inapplicable (NaN), further validating the model’s ability to identify patients who truly do not benefit from MT (Table S2).

#### Temporal stability

We stratified the cohort into early (*n* = 31), mid (*n* = 145), and late (*n* = 248) eras to rule out era-specific practice shifts over the nearly 2-decade study period. Prognostic predictive power remained consistently high across all periods (early AUROC = 0.970, 95% CI [0.895, 1.000]; mid AUROC = 0.929, 95% CI [0.883, 0.966]; late AUROC = 0.920, 95% CI [0.879, 0.951]). Crucially, treatment effect heterogeneity persisted remarkably over time: the AUQC for all eras remained highly negative (early = −0.95, 95% CI [−1.77, −0.06]; mid = −7.21, 95% CI [−9.70, −4.26]; late = −16.35, 95% CI [−19.30, −12.80]). The benefit differences between the highest and lowest quartiles were consistently negative and significant across all eras (early diff = −0.917, 95% CI [−2.000, −0.184]; mid diff = −1.575, 95% CI [−1.900, −0.963]; late diff = −1.335, 95% CI [−1.637, −1.037]), with all 95% CIs cleanly excluding zero despite varying sample sizes and historical MT adoption rates. This demonstrates that our model captures stable, intrinsic biological effect modifiers that are robust to historical practice evolution (Fig. S5).

## Discussion

In this study, we demonstrate that a machine learning framework can deconstruct the heterogeneous benefit of maintenance rituximab (MT) in FL, providing a data-driven path toward personalized therapy. By moving beyond traditional risk prediction, our DML model estimates the ITE for each patient, effectively distinguishing those who are highly likely to benefit from MT from those who are not. A key finding from our retrospective analysis is the identification of a “low-risk, low-benefit” subgroup that might be potential candidates to be observed without MT. This work provides a powerful proof of concept for optimizing the risk–benefit calculus in oncology.

### Contextualizing findings within the academic literature

The current standard of 2 years of MT following induction therapy, established largely by the Primary Rituximab and Maintenance (PRIMA) trial [19], applies a “one-size-fits-all” approach that universally exposes patients to potential overtreatment, financial toxicity, and increased infection risks. The oncology community has urgently sought to address this through risk-adapted postinduction strategies. For instance, the FOLL12 trial attempted to use metabolic response (positron emission tomography [PET]–computed tomography) and MRD status to guide MT omission. However, omitting MT based solely on negative PET/MRD status resulted in inferior progression-free survival compared to that of standard MT.

This paradox highlights a fundamental flaw in current clinical paradigms: traditional prognostic markers (like PET status or the FLIPI score) identify a patient’s baseline risk but are not fundamentally predictive of treatment benefit. A low-risk patient might still derive substantial relative benefit from MT to eradicate microscopic disease. Our study bridges this critical gap. By applying causal inference techniques to observational data, our framework explicitly calculates the counterfactual—estimating the magnitude of risk reduction directly attributable to MT for a specific patient. This evolution from prognostic risk stratification to predictive benefit estimation offers a methodological blueprint for future risk-adapted trials.

### Clinical implications and rethinking “model-aligned” care

The most important clinical implication of our work is the data-driven challenge to universal MT. In our retrospective simulation, patients in the model-identified “low-risk, low-benefit” group who withheld MT experienced zero progressions, standing in sharp contrast to the guideline-based equivalent group where 20% of observed patients progressed. Furthermore, alignment between actual clinical decisions and our model’s recommendations was associated with a lower progression rate (14.8% vs. 38.0%).

However, we must interpret this alignment-based outcome with necessary caution. Treatment alignment in this retrospective cohort is a post hoc construct driven by historical clinician behavior rather than a randomized exposure. It inherently captures unmeasured clinical intuition—such as subtle variations in performance status, patient preference, or care quality—that may confound the observed outcomes. Therefore, rather than viewing these results as definitive evidence of algorithmic superiority over clinical guidelines, they should be viewed as compelling, hypothesis-generating evidence. By making the risk–benefit trade-off mathematically transparent, our framework provides a refined tool to augment, rather than replace, shared clinical decision-making.

### Methodological robustness and temporal stability

A major strength of our study is the rigorous validation of the causal framework. To ensure that our estimates were not artifacts of algorithmic extrapolation, we incorporated target trial emulation principles and verified substantial propensity score overlap (common support) between the MT and non-MT groups. Furthermore, the FL treatment landscape has evolved over the nearly 2-decade study period (2002 to 2019). Our temporal stratification analysis demonstrated that the model’s discriminative power and the identified treatment effect heterogeneity remained highly stable across early, mid, and late eras. This confirms that the model captured stable, biology-driven effect modifiers rather than merely proxying historical practice shifts. Finally, our E value sensitivity analysis quantified the robustness of our findings against unmeasured confounding, demonstrating that the substantial benefit observed in the top-quartile patients would require remarkably strong, unmeasured confounders to be fully negated.

### Limitations

This study has several important limitations. The primary limitation is its retrospective, single-center design, which lacks independent external validation. Although we employed nonparametric bootstrapping and E-value analyses to mitigate confounding and variance, we cannot entirely rule out the influence of unmeasured variables. Specifically, the low-benefit estimations remain sensitive to unmeasured confounders, highlighting the need to incorporate deeper molecular profiles (e.g., m7-FLIPI mutational status) and socio-economic determinants in future model iterations.

Secondly, a comprehensive clinical risk–benefit analysis must extend beyond our primary endpoint of POD24. Due to the retrospective nature of the electronic health record data, we lacked granular, systematic tracking of long-term toxicities (e.g., Common Terminology Criteria for Adverse Events grading), infection-related hospitalizations, and cumulative healthcare billing records [20]. To address this gap, we performed a supplemental literature-based projection and cost estimation (Supplementary Materials A.2). This analysis indicated that our model’s recommendations might lead to a modest increase in upfront treatment burden, estimated drug costs, and projected adverse events compared to standard guidelines, driven by a slightly broader recommendation for MT (68.0% vs. 61.7% of patients). However, this upfront investment must be weighed against the substantial long-term clinical and economic benefits of preventing POD24. Ultimately, the true net clinical benefit of our proposed interactive decision-support tool must be validated in a prospective, randomized clinical trial with rigorous pharmacoeconomic endpoints, which remains the gold standard for establishing practice-changing evidence.

More broadly, the integration of traditional statistical methods with machine learning approaches represents a promising direction for strengthening causal inference from observational clinical data [21], and future iterations of our framework could benefit from such hybrid methodologies.

## Conclusion

In conclusion, this study introduces a robust DML framework to personalize MT in FL. By disentangling baseline prognostic risk from individualized predictive benefit, we identified specific patient subgroups that gain substantial advantage from continued therapy, as well as those who might safely forgo it. Supported by an interactive web-based tool incorporating uncertainty quantification, this approach offers a translational template to minimize overtreatment, reduce healthcare burdens, and advance the paradigm of precision oncology. Prospective clinical trials are warranted to validate the real-world impact of this model-guided strategy.

## Ethical Approval

The study protocol was reviewed and approved by the Institutional Review Board or independent ethics committee of each participating institution (2025KT13 and 2025KT74).

## Data Availability

The clinical dataset analyzed during the current study may be made available from the corresponding authors upon reasonable request and subject to approval from the institutional review boards of the participating centers. The analysis code used for the model implementation, data analysis, and figure generation is available in a public repository at https://github.com/v1xerunt/DML-Lymphoma. The online prediction tool is publicly available at https://fl-ite.medx-pku.com.
