# Supplementary material for: Personalizing Maintenance Rituximab in Follicular Lymphoma: A Machine Learning Framework for Risk–Benefit Optimization
Source: Health Data Sci. 2026 Aug 12;6:0467. doi: 10.34133/hds.0467 (PMC13462244; doi:10.34133/hds.0467)
Supplement: Supplementary file 1 [file hds.0467.f1.zip › Supplementary Materials.pdf]

A Supplementary Materials

A.1 Clinical Guideline Recommendations for Maintenance Therapy

The following criteria were used to decide whether a patient should receive maintenance therapy or not in clinical practice based on the guideline recommendation[16].

- Any lymph node or extranodal mass with a diameter $\geq$ 7cm.
- $\geq$ 3 involved lymph nodes with a diameter $\geq$ 3cm.
- Symptomatic splenomegaly.
- B symptoms.
- Pleural or peritoneal effusion.
- White blood cell count $< 1.0 \times 10^9/L$ and/or platelet count $< 100 \times 10^9/L$.
- Lymphoma leukemia.

A.2 Estimation of Treatment Burden, Projected Safety, and Costs

To address the absence of individual-level toxicity and economic data in our retrospective cohort, we employed a projection strategy to compare the Model-Recommended strategy (guided by our SVM framework) against a Guideline-Concordant strategy (MT recommended based on high tumor burden criteria, Appendix 13.1). Treatment burden was estimated assuming a standard 2-year maintenance protocol (375 mg/m^2 every 2 months for 2 years, totaling 12 infusions per patient). Expected adverse events (AEs) were projected by mapping the treatment exposure proportions of both strategies to the AE incidence rates reported in the landmark PRIMA trial (e.g., Grade $\geq$ 3 infection rates of 4% for MT and 1% for observation). Treatment costs (drug acquisition only) were estimated across multiple pricing scenarios, including Originator and Biosimilar pricing in the Chinese National Reimbursement Drug List (NRDL) and US markets. Cost differences between the Guideline and Model strategies were quantified with 95% confidence intervals (CIs) generated via 2,000 non-parametric bootstrap resamples to account for subgroup variance.

The agreement rate between the Model-Recommended and Guideline-Concordant strategies was 62.0%. The Model strategy recommended MT for 68.0% (279/404) of patients, whereas the Guideline strategy recommended MT for 61.7% (253/404). Consequently, the Model strategy resulted in an estimated 10.3% increase in total infusions (+312 infusions cohort-wide). As detailed in Table S1, this broader coverage correspondingly led to modestly higher projected costs and adverse events. Under the primary evaluation scenario (Originator NRDL pricing at 8,000 CNY/infusion), the Model strategy yielded a mean estimated cost of 65,327 CNY per patient versus 59,239 CNY for the Guideline strategy (Bootstrap B=2,000 mean difference: -6,171 CNY, 95% CI [-11,947, -468]). Safety projections indicated approximately +0.8 expected Grade $\geq$ 3 infections and +0.9 Grade $\geq$ 3 neutropenia events in the Model arm compared to the Guideline arm (Figure S1). These findings suggest a slight increase in upfront treatment burden to achieve the model's optimized POD24 control.

Table S1. Comparison of Treatment Burden, Estimated Costs, and Projected Safety between Model-Recommended and Guideline-Concordant Strategies.

Category	Metric	Model-Recommended	Guideline-Concordant	Difference (Model − Guideline)
**Recommendation Overlap**	Agreement rate	62.0%		—
	Both recommend MT	188		—
	Model yes, Guideline no	91		—
	Model no, Guideline yes	65		—
	Both recommend no MT	66		—
**Treatment Burden**	Patients receiving MT	279 (68.0%)	253 (61.7%)	+26 (+6.3%)
	Total estimated infusions	3,348	3,036	+312 (+10.3%)
**Estimated Cost (Per-Patient)**	CN Originator (NRDL, CNY)	65,327	59,239	+6,088
	CN Biosimilar (NRDL, CNY)	16,332	14,810	+1,522
	US Insured (avg, USD)	32,663	29,620	+3,044
**Projected Safety (Events)**	Grade $\geq$3 infection	12.5	11.7	+0.8
	Grade $\geq$3 neutropenia	11.8	10.9	+0.9
	Any-grade infection	140.2	136.3	+3.9
	Any-grade neutropenia	113.4	107.4	+6.0

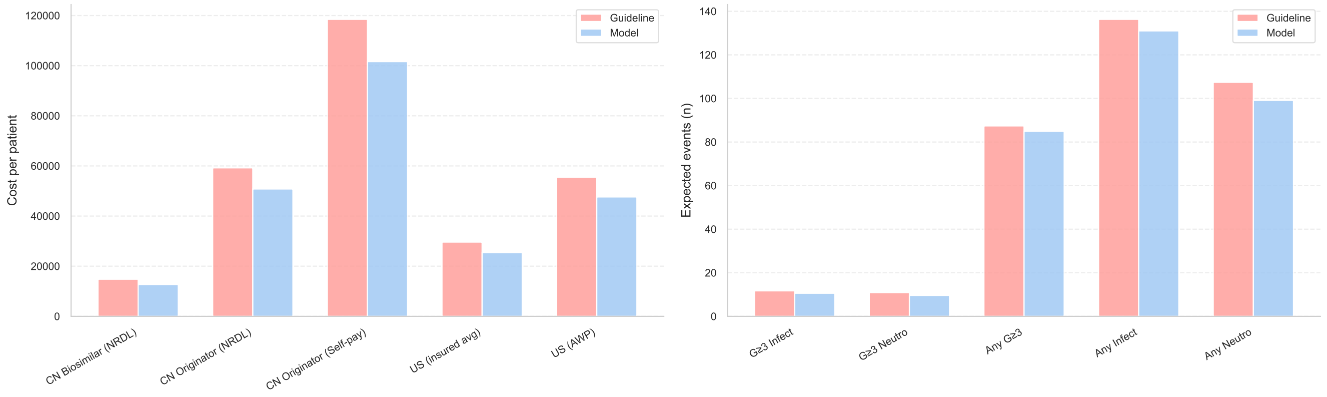

Figure S1. Economic, Safety, and Treatment Burden Implications of the Personalized Recommendation Framework. (a) Estimated Treatment Costs. Comparison of projected per-patient drug acquisition costs across five real-world pricing scenarios, including the Chinese National Reimbursement Drug List (NRDL) for originator and biosimilar rituximab, as well as US insured and average wholesale price (AWP) estimates. The model-recommended strategy incurs a modest increase in upfront costs across all scenarios due to a broader allocation of maintenance therapy (MT). **(b) Projected Adverse Events.** Expected safety events extrapolated by mapping the cohort's treatment exposure under each strategy to the incidence rates reported in the PRIMA trial. The model's recommendations result in a marginal increase in expected Grade $\geq$3 infections and neutropenia.

A.3 Supplementary Tables

Table S2. E-value Sensitivity Analysis for Unmeasured Confounding Across Benefit Quartiles. This table presents the relative risk (RR) and corresponding E-values for patients stratified into quartiles based on their predicted individualized treatment effect (ITE), where Q1 represents the highest predicted benefit and Q4 the lowest. The *Point E-value* represents the minimum strength of association (on the risk ratio scale) an unmeasured confounder must have with both treatment assignment and the POD24 outcome to completely explain away the observed point estimate of the MT protective effect. The *Conservative E-value Bound* is calculated based on the confidence interval limit closest to the null (RR=1). Note: The overall individual E-value median is 2.09. For Q4, because the observed RR is $>$ 1 (indicating no protective effect), the E-value formula is inherently inapplicable (denoted as NaN), further validating the lack of benefit in this subgroup.

Subgroup	RR Point Estimate	RR 95% CI	Point E-value	Conservative E-value Bound
Q1 (Highest Benefit)	0.61	[0.54, 0.70]	2.68	2.23
Q2	0.64	[0.57, 0.76]	2.49	1.95
Q3	0.92	[0.79, 0.98]	1.38	1.18
Q4 (Lowest Benefit)	1.28	[1.08, 1.37]	-	-

A.4 Supplementary Figures

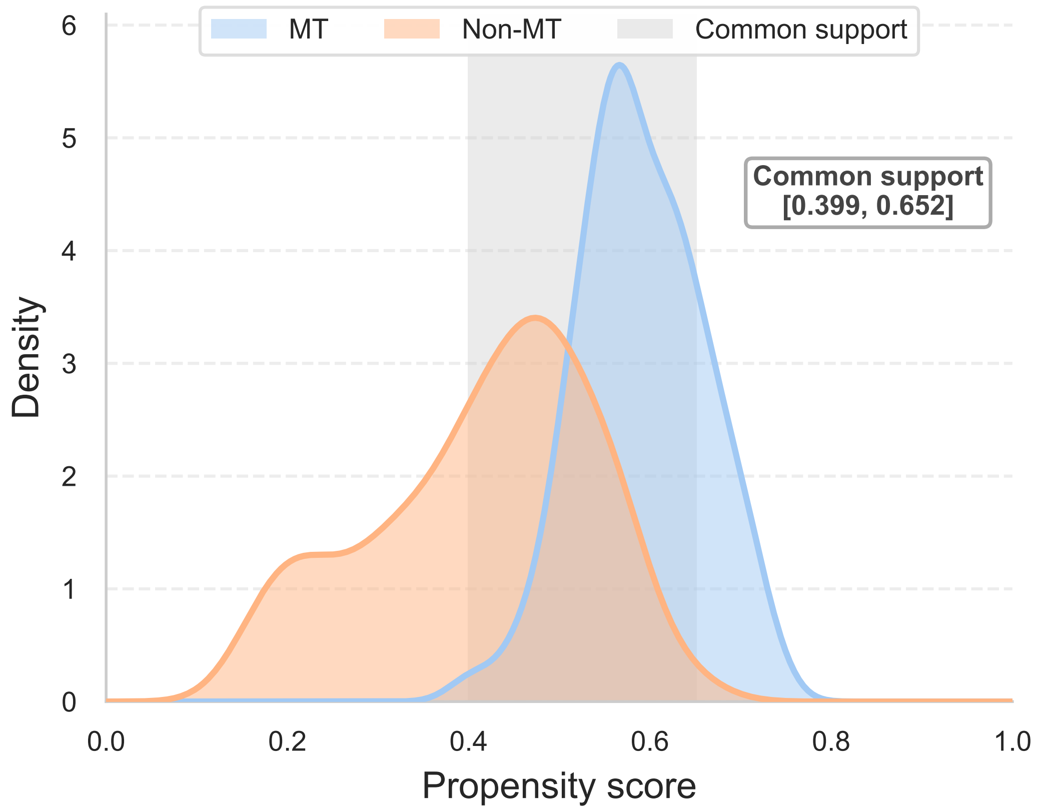

Figure S2. **Propensity Score Overlap and Common Support Density Plot.** Distribution of estimated propensity scores for receiving maintenance rituximab (MT) among the observed MT group (blue) and non-MT group (orange). The shaded gray area represents the defined "common support" region [0.399, 0.652], capturing 69.8% of the patient cohort. This overlap confirms sufficient clinical equipoise within the study population, ensuring that our double machine learning causal inferences are not heavily reliant on algorithmic extrapolation.

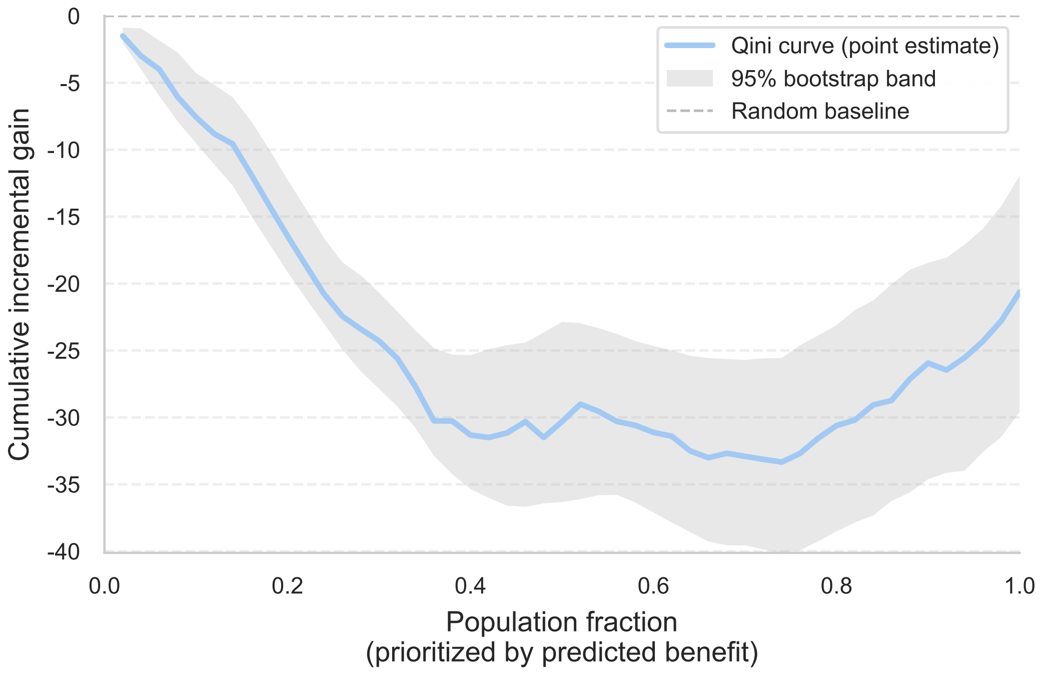

Figure S3. Bootstrap Qini Curve with 95% Confidence Interval Band. The Qini curve illustrates the cumulative incremental gain of utilizing the double machine learning (DML) model to prioritize maintenance therapy based on the predicted individualized treatment effect (ITE). The solid blue line represents the point estimate from the full sample, while the shaded blue area indicates the 95% confidence interval (CI) band generated via non-parametric bootstrapping with B=2,000 resamples. The dashed gray line represents a random allocation strategy.

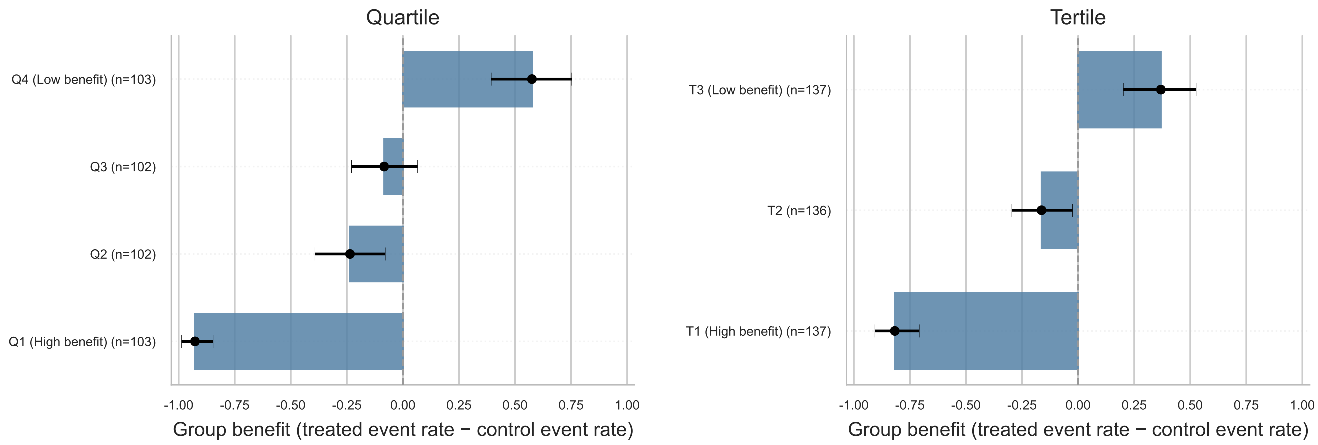

Figure S4. Empirical Benefit Differences Across Quantile-Stratified Subgroups. Forest plot depicting the point estimates and 95% confidence intervals (CIs) for the empirical treatment benefit (treated event rate minus control event rate) within quantile-stratified subgroups. Patients were stratified into quartiles and tertiles based on their DML-predicted individualized treatment effect (ITE). Negative benefit values indicate a reduction in the risk of POD24 (disease progression within 24 months) attributable to MT. The 95% CIs were derived from non-parametric bootstrapping (B=2,000 resamples). The significant separation and non-overlapping CIs between the highest-benefit (e.g., Q1) and lowest-benefit (e.g., Q4) groups robustly validate the model's capacity to identify true responders versus non-responders.

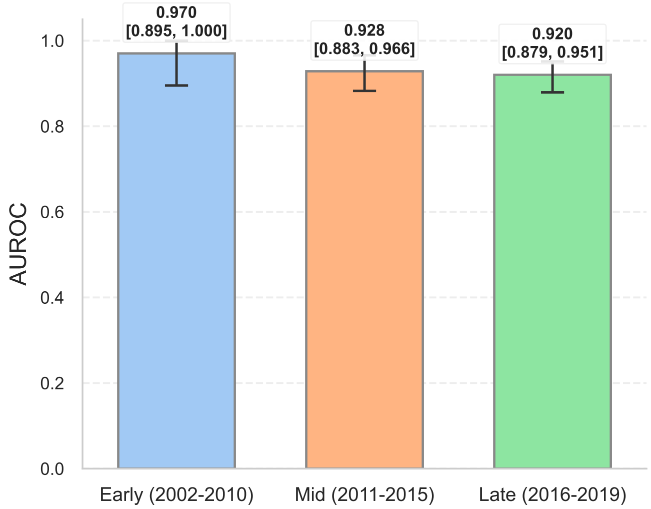

(a) AUROC

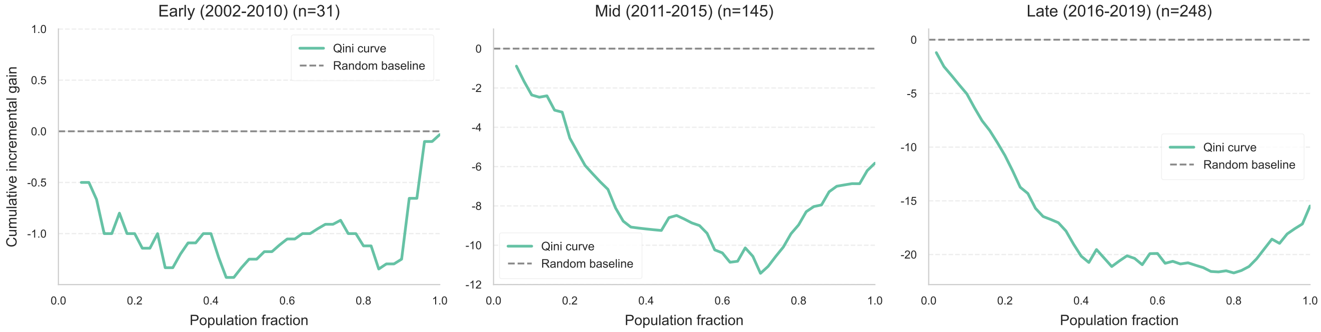

(b) Qini Curves

Figure S5. Temporal Stability of Predictive Performance and Treatment Heterogeneity Across Eras. Evaluation of the model's temporal robustness by stratifying the study cohort (20022019) into three eras: Early (20022010, n=31), Mid (20112015, n=145), and Late (20162019, n=248). (A) Bar chart showing the consistent prognostic predictive performance (AUROC) of the model across the three eras, with error bars representing 95% CIs from B=2,000 bootstrap resamples. (B-D) Qini curves for the Early, Mid, and Late eras, respectively. The consistent divergence of the Qini curves from the random baseline across all time periods demonstrates that the captured treatment effect heterogeneity reflects stable, biology-driven modifiers rather than era-specific shifts in clinical practice.
