# Supplementary material for: Personalizing Maintenance Rituximab in Follicular Lymphoma: A Machine Learning Framework for Risk–Benefit Optimization
Source: Health Data Sci. 2026 Aug 12;6:0467. doi: 10.34133/hds.0467 (PMC13462244; doi:10.34133/hds.0467)
Supplement: Supplementary file 1 [file hds.0467.f1.zip › figure_s1.pdf]

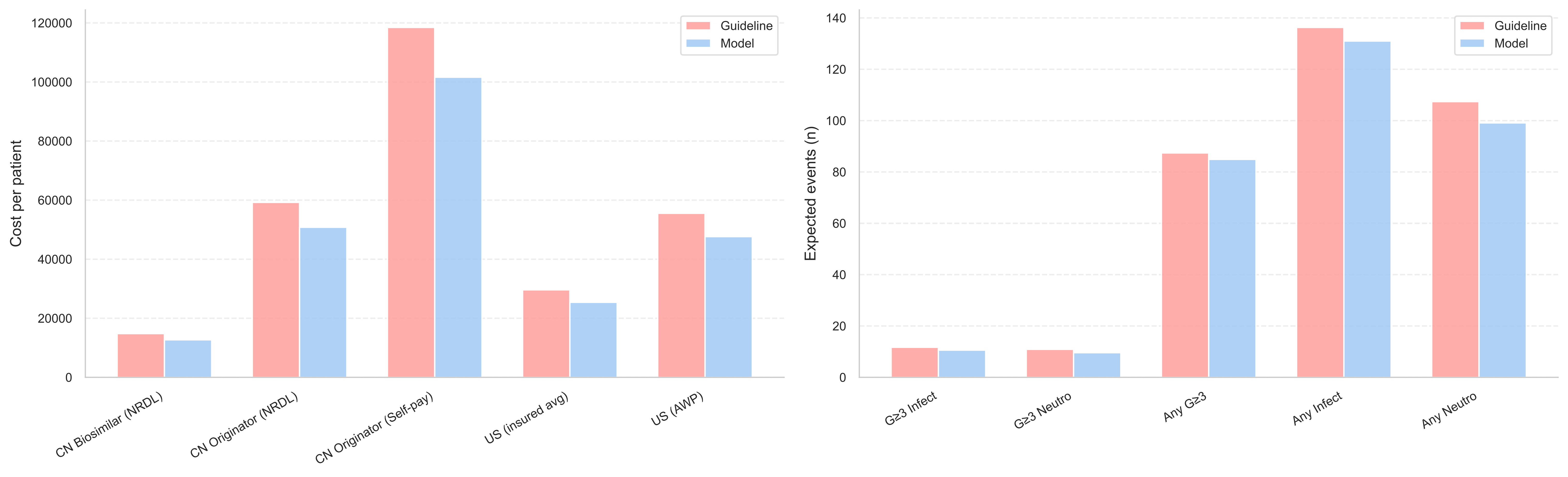

Guideline
Model
Cost per patient
120000
100000
80000
60000
40000
20000
0
CN Biosimilar (NRDL)
CN Originator (NRDL)
CN Originator (Self-pay)
US (insured avg)
US (AWP)
Expected events (n)
140
120
100
80
60
40
20
0
G≥3 Infect
G≥3 Neutro
Any G≥3
Any Infect
Any Neutro
