# Supplementary material for: Personalizing Maintenance Rituximab in Follicular Lymphoma: A Machine Learning Framework for Risk–Benefit Optimization
Source: Health Data Sci. 2026 Aug 12;6:0467. doi: 10.34133/hds.0467 (PMC13462244; doi:10.34133/hds.0467)
Supplement: Supplementary file 1 [file hds.0467.f1.zip › figure_s2.pdf]

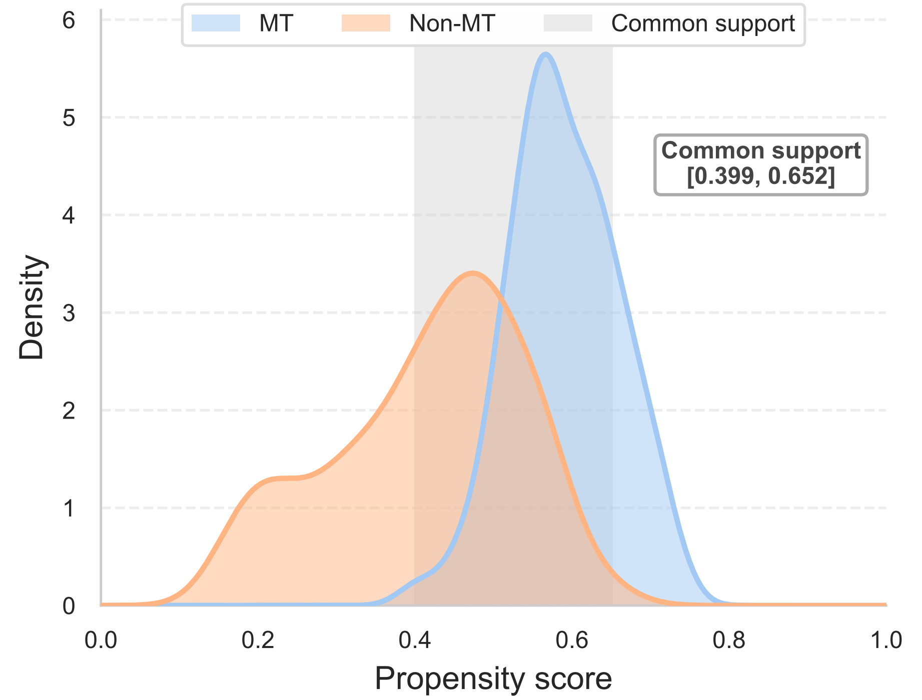

MT
Non-MT
Common support
Common support
[0.399, 0.652]
Density
Propensity score
0
1
2
3
4
5
6
0.0
0.2
0.4
0.6
0.8
1.0
