# Supplementary material for: Personalizing Maintenance Rituximab in Follicular Lymphoma: A Machine Learning Framework for Risk–Benefit Optimization
Source: Health Data Sci. 2026 Aug 12;6:0467. doi: 10.34133/hds.0467 (PMC13462244; doi:10.34133/hds.0467)
Supplement: Supplementary file 1 [file hds.0467.f1.zip › figure_s3.pdf]

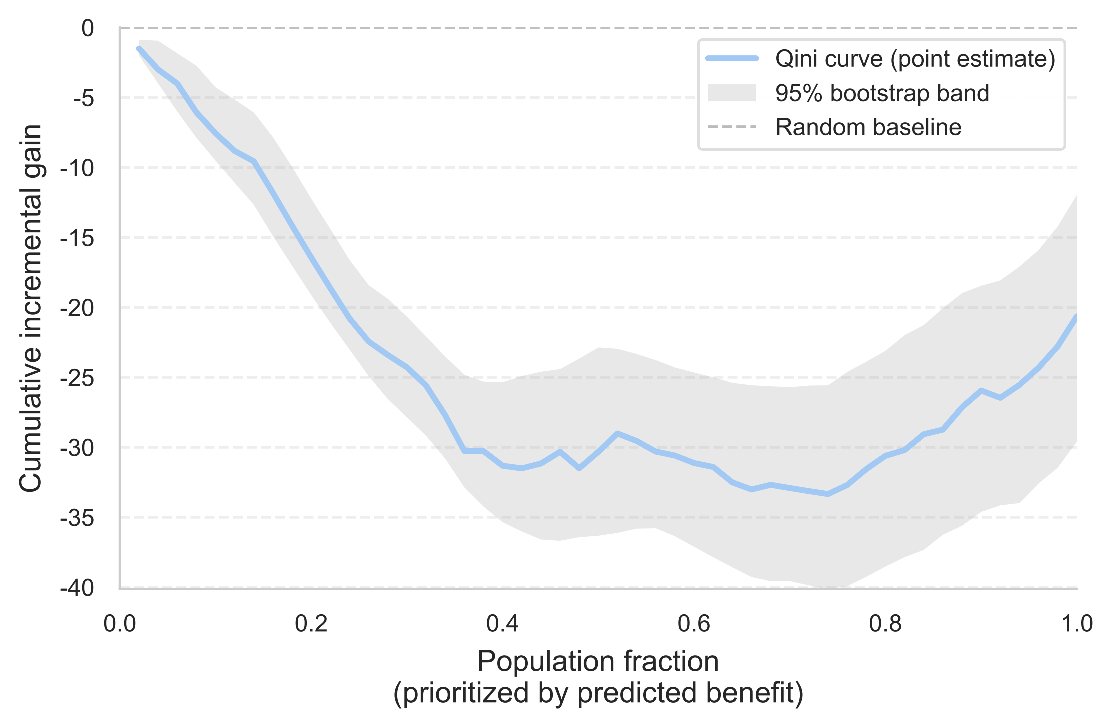

Qini curve (point estimate)
95% bootstrap band
Random baseline
Cumulative incremental gain
0
-5
-10
-15
-20
-25
-30
-35
-40
0.0
0.2
0.4
0.6
0.8
1.0
Population fraction
(prioritized by predicted benefit)
