# Supplementary material for: Personalizing Maintenance Rituximab in Follicular Lymphoma: A Machine Learning Framework for Risk–Benefit Optimization
Source: Health Data Sci. 2026 Aug 12;6:0467. doi: 10.34133/hds.0467 (PMC13462244; doi:10.34133/hds.0467)
Supplement: Supplementary file 1 [file hds.0467.f1.zip › figure_s4.pdf]

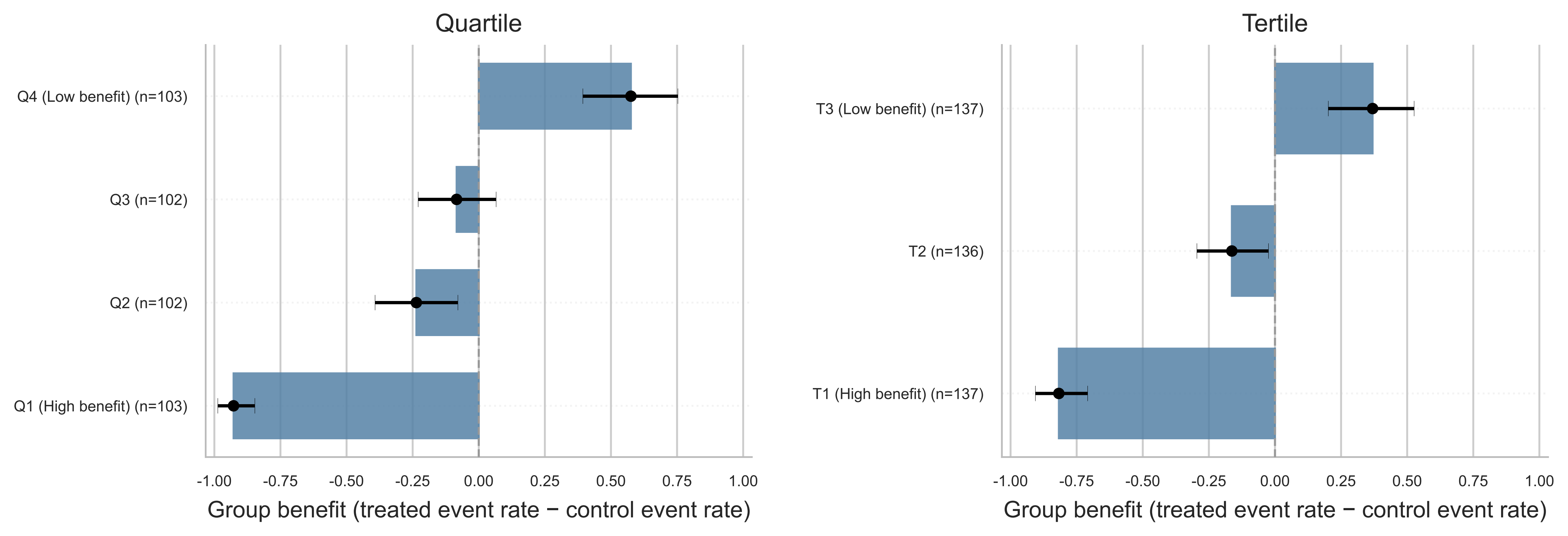
Quartile
Q4 (Low benefit) (n=103)
Q3 (n=102)
Q2 (n=102)
Q1 (High benefit) (n=103)
-1.00
-0.75
-0.50
-0.25
0.00
0.25
0.50
0.75
1.00
Group benefit (treated event rate − control event rate)
Tertile
T3 (Low benefit) (n=137)
T2 (n=136)
T1 (High benefit) (n=137)
-1.00
-0.75
-0.50
-0.25
0.00
0.25
0.50
0.75
1.00
Group benefit (treated event rate − control event rate)
