# Supplementary material for: Personalizing Maintenance Rituximab in Follicular Lymphoma: A Machine Learning Framework for Risk–Benefit Optimization
Source: Health Data Sci. 2026 Aug 12;6:0467. doi: 10.34133/hds.0467 (PMC13462244; doi:10.34133/hds.0467)
Supplement: Supplementary file 1 [file hds.0467.f1.zip › figure_s5a.pdf]

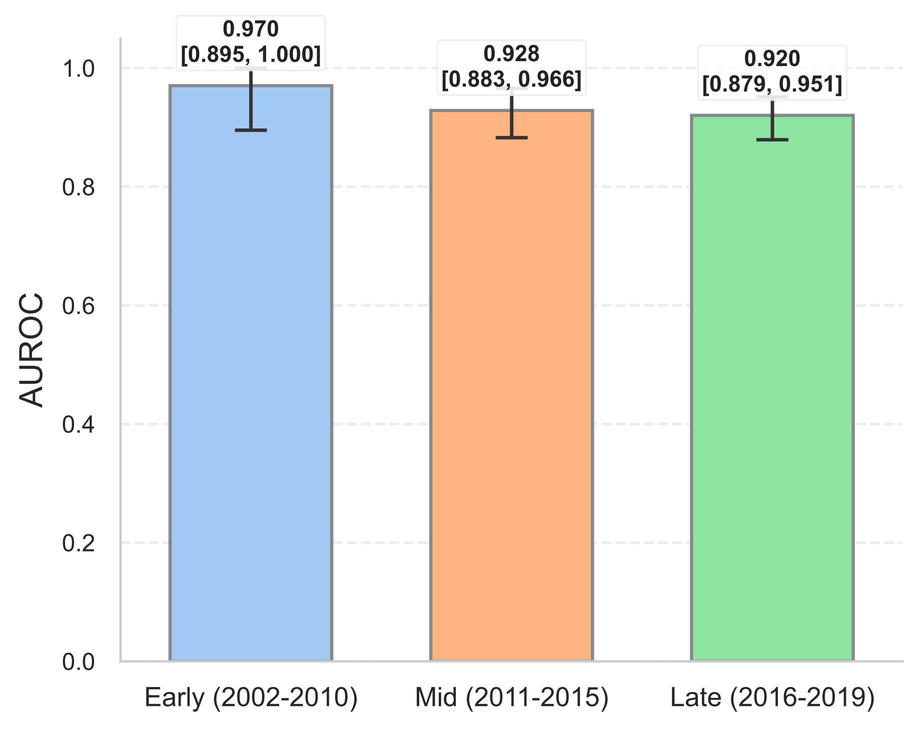
0.970
[0.895, 1.000]
0.928
[0.883, 0.966]
0.920
[0.879, 0.951]
AUROC
1.0
0.8
0.6
0.4
0.2
0.0
Early (2002-2010)
Mid (2011-2015)
Late (2016-2019)
