# Supplementary material for: Personalizing Maintenance Rituximab in Follicular Lymphoma: A Machine Learning Framework for Risk–Benefit Optimization
Source: Health Data Sci. 2026 Aug 12;6:0467. doi: 10.34133/hds.0467 (PMC13462244; doi:10.34133/hds.0467)
Supplement: Supplementary file 1 [file hds.0467.f1.zip › figure_s5b.pdf]

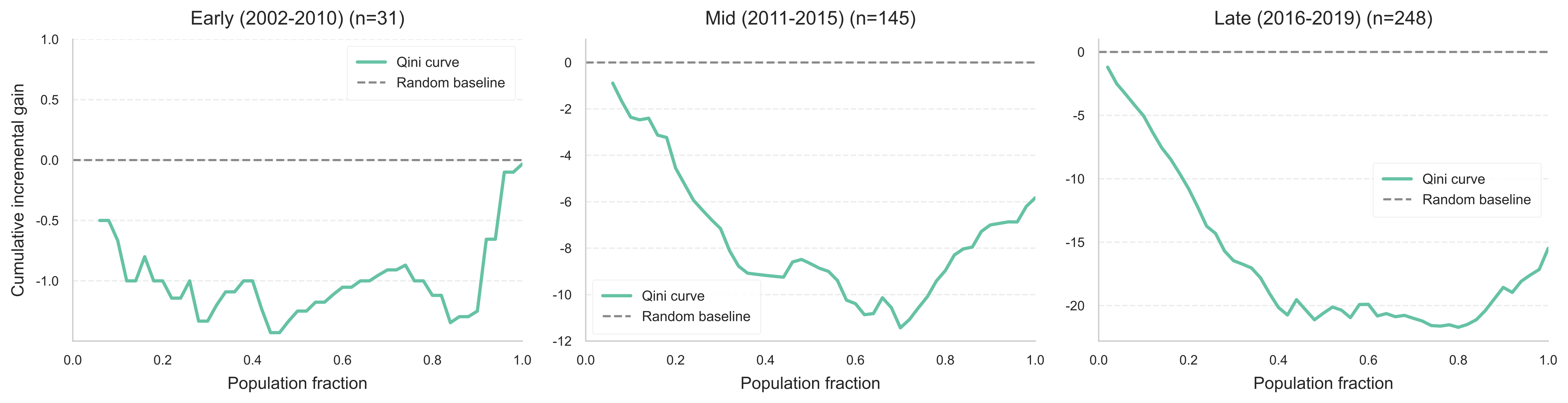
Early (2002-2010) (n=31)
Mid (2011-2015) (n=145)
Late (2016-2019) (n=248)
Cumulative incremental gain
Population fraction
Qini curve
Random baseline
1.0
0.5
0.0
-0.5
-1.0
0
-2
-4
-6
-8
-10
-12
-5
-15
-20
0.0
0.2
0.4
0.6
0.8
1.0
